# Clustering infection of hepatitis B virus genotype B4 among residents in Vietnam, and its genomic characters both intra- and extra-family

**DOI:** 10.1371/journal.pone.0177248

**Published:** 2017-07-28

**Authors:** Junko Matsuo, Son Huy Do, Chikako Yamamoto, Shintaro Nagashima, Channarena Chuon, Keiko Katayama, Kazuaki Takahashi, Junko Tanaka

**Affiliations:** 1 Department of Epidemiology, Infectious Disease Control and Prevention, Institute of Biomedical and Health Sciences, Hiroshima University, Hiroshima, Japan; 2 Binh Thuan Medical College, Phan Thiet City, Binh Thuan Province, Vietnam; 3 Department of Medical Sciences, Toshiba General Hospital, Tokyo, Japan; Centre de Recherche en Cancerologie de Lyon, FRANCE

## Abstract

Vietnam has a high rate of hepatitis B virus (HBV) infection and a high mortality rate from hepatocellular carcinoma. We performed a detailed genetic analysis of 48 residents and four families from Binh Thuan Province, a southern coastal area of Vietnam. The route of infection and genomic characteristics related to hepatocellular carcinoma (HCC) were studied in HBV spread among carriers that we detected in our previous hepatitis survey. The HBV genotype was B4 in 91.7% and C1 in 8.3% of the cases. The intra-family’s HBV sequence homology was high at 96.8–99.4%. However, it was also high at 99.4–99.8% among residents of the same age and sex as family members. In addition, full genome analysis was performed in 21 cases. The core region of all 20 isolates with genotype B4 was a recombinant of genotype C, and pre-S deletion was found in 20% of cases. The promoter mutation G1613A was found in 13.6% of cases, and a 24 bp insertion from nt1673 in the X region was found in 6.3% of cases. The phylogenetic tree and homology analysis of the HBV full genome suggested the probability and its possibility of horizontal transmission not only within families nor vertical transmission but within cohorts of the same generation in the population. Moreover, the HBV genotype B4 isolates were found not only to be recombinants of genotype C, which results in a high cancer risk, but also to have other risk of HCC, pre-S deletions, the G1613A mutation, and X region insertions corresponding to the promoter. These genomic characters were suggested to be one of the factors to explain the high HCC mortality rate in Vietnam.

## Introduction

There are an estimated 240 million hepatitis B virus (HBV) carriers around the world, and 686,000 people reportedly die of liver diseases including cirrhosis and liver cancer caused by HBV infection every year [1]. The World Health Organization (WHO) recommends universal vaccination with a HB vaccine as a preventative measure for HBV infection, which has been implemented in various countries. However, there are still many HBV carriers in Asia and Africa, a significant source of future cirrhosis and hepatocellular carcinoma (HCC) cases.

Main reasons of HBV carriers are believed to be vertical transmission during perinatal period from mothers to babies, or horizontal transmission during early childhood when immuno-competence is not fully developed. It is known that intra-family clustering is common in the high endemic areas of Southeast Asia, and that what is more to the primary vertical transmission route, there is also a high risk of horizontal transmission between intra-familial cohabitants [2, 3].

In Africa, there are areas where vertical transmission occurs at a lower rate than in Southeast Asia and horizontal transmission in early childhood is the primary transmission route [4–6]. This is considered to be potentially arisen from the differences in genotype; while genotypes A, D and E are predominant in Africa, genotypes B and C are the dominant genotypes in Southeast Asia [7, 8].

The Socialist Republic of Vietnam is known to be a highly endemic area for HBV [1, 9–11]. Thus, we performed a pilot study on hepatitis in 2012 with the general population of Vietnam as our subjects. We found that hepatitis B surface antigen (HBsAg)-positive rate was high at 15.3%. HBV genotype B was dominant, at about 80% of cases [12]. Furthermore, in order to investigate transmission pathways in the same population, we performed a family study with individuals identified as HB carriers that were referred to as index persons. HBV genotype B is known to be associated with lower HCC risk than genotype C [13–16]. However, according to the WHO, Vietnam is an area where HCC is prevalent, ranking fourth in liver cancer deaths [17]. There are numerous reports indicating that not only HBV genotypes, promoter mutations and pre-S deletions are both risk factors for HCC [18, 19]. Therefore, in the present study we performed detailed genetic analysis of HBV found during the pilot study, with the aim to investigate the characteristics of HBV isolates and the transmission pathways of HBV.

## Materials and methods

### Study design

In 2012, we performed a population-based hepatitis survey by randomly selecting 170 residents from each of three randomly selected wards in Binh Thuan Province with a population of 10,000 people. It is located on the coast about 200 km away from Ho Chi Minh City. A total of 509 subjects were enrolled. Then we performed a family survey of the families of the HBsAg-positive carriers that we found [9].

**Fig 1:**
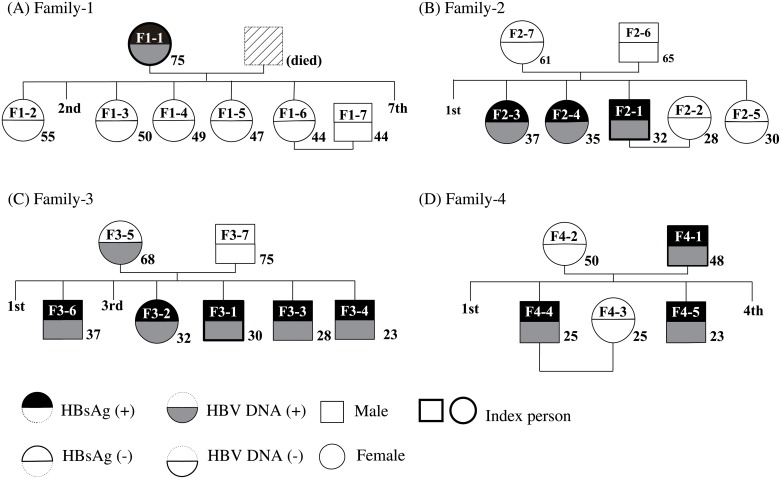
Family trees of family members with their status of HBsAg and HBV DNA. A) Family 1, B) Family 2, C) Family 3, D) Family 4. The index persons are indicated with bold rim. Their age at the study conducted are shown. We could detect HBV DNA in 13 cases with nested PCR for sequence.

In the present study, we performed genetic analysis of HBV with stored blood sera of 4 individuals as index persons who agreed to invest their family members (Fig 1), and more 35 residents of which sera could be amplified PCR product for HBV sequence out of 77 individuals in general population who were found to have an HBsAg positive. Additionally, we subjected 22 family members of four index person’s families, as 26 members in total in the four family trees. (Table 1). Age distribution ranged 21–75 years old in the 61 total participants.

**Table 1:** Demographic characteristics of residents and family members.

Participants	Total	Residents including four index persons	Family members for four index persons
n	61	39	22
Men / Female	31/ 30	23/ 16	8/ 14
Mean of age (years)	38.2± 13.5	36.6± 12.7	41.0± 14.3
Median (years)	32.0	30.0	37.0
Range (years)	21–75	21–75	23–75

### Ethical permission

This study was approved by the Ethics Committees for epidemiological research of Hiroshima University (permission number 507–2) in Japan and the Binh Thuan Provincial Department of Health in Vietnam. All participants were adults. Details on benefits and risks of this study were explained and written informed consent was obtained from each participant.

### Partial sequencing

HBV DNA was extracted from 100 μl samples of stored blood serum using SMITEST EX-R&D^®^ (Genome Science Laboratories, Fukushima, Japan) and then nested PCR was performed using Prime STAR^®^ GXL polymerase (Takara Bio Inc., Shiga, Japan), the primer set WA-L and WA-R [20] and the inner primers WA-L2 and WA-R2 (Table 2). The obtained PCR product (fragment A) was directly sequenced using the primers WA-2R, FA2R, FA4L [20], and B1260 (Table 2) to determine the nucleotide sequence at the nt715-1482 and nt1600-1790 sites. Samples were sequenced with Big-Dye Terminator v3.1 Cycle Sequencing Kit (Thermo Fisher Scientific K.K., Kanagawa, Japan) and defined using auto-sequencer, Applied Biosystems 3730xl DNA Analyzer (Thermo Fisher Scientific K.K., Kanagawa, Japan).

**Table 2:** Primers for PCR and sequencing.

name	nucleotide number	direction	sequence (5' to 3')
***fragment A***			
WA-L*	1862–1885	sense	ACTGTTCAAGGGTCCAAGCTGTGC
WA-R*	1806–1829	antisense	AGCAAAAAGTTGCATGGTGCTGGT
FA2R*	217–240	antisense	GGTATTGTGAGGADDYTTGTCAAC
FA4L*	801–820	sense	GTATTGGGGGCCAAGTCTGT
FA4LAS	801–820	antisense	ACAGACTTGGCCCCCAATAC
FA3L*	107–124	sense	CTGCTGGTGGCTCCAGTT
WA-2R	1781–1802	antisense	CAGACCAATTTATGCCTACAGC
WA-2L	1887–1908	sense	GGTGGCTTTRGGRCATGGACAT
P4AS	455–474	antisense	CAAGGTTATGTTGCCCGTTTG
B260	260–279	antisense	AGAAAATTGAGAGAAGTCCA
B1260	1260–1279	sense	GCCGATCCATACTGCGGAAC
B2466	2466–2485	antisense	GTAAAGTTTCCCACCTTATG
B2830	2830–2849	antisense	ATGCTGTAGCTCTTGTTCCC
***fragment B***			
S1	1414–1434	sense	ACGTCCTTTGTTTACGTCCCG
S2	1436–1456	sense	CGGCGCTGAATCCCGCGGACG
S3	1489–1508	sense	CCGCTTCTCCGTCTGCCGTA
S4	1527–1547	sense	CACCTCTCTTTACGCGGACTC
AS1	2130–2110	antisense	TCCAAATTACTTCCCACCCAG
AS2	2160–2140	antisense	CTGACTACTAATTCCCTGGAT
AS3	2185–2165	antisense	TAGGCCCATATTAACATTGAC
AS4	2098–2078	antisense	CATCAACTCACCCCAACACAG
***mtDNA***			
mtDNA-1F	15932–15953	sense	GAGATGAAACCTTTTTCCAAG
mtDNA-2F	15956–15979	sense	CAAATCAGAGAAAAAGTCTTTAAC
mtDNA-3F	15982–16001	sense	CACCATTAGCACCCAAAGCT
mtDNA-1R	503–521	antisense	TGTGTGCTGGGTAGGATGG
mtDNA-2R	436–456	antisense	AAATAATGTGTTAGTTGGGGG
mtDNA-3R	410–431	antisense	CTGTTAAAAGTGCATACCGCCA

*: primers are reported previously in reference 20.

mtDNA, mitochondrial DNA.

### Mitochondria analysis

The DNA extracted from the 100 μl samples of stored blood serum using SMITEST EX-R&D^®^ (Genome Science Laboratories, Fukushima, Japan) was subjected to nested PCR using Prime STAR^®^ GXL polymerase and a primer set targeting the mitochondrial hypervariable region (HVR), namely mtDNA-1F, mtDNA-2F, mtDNA-1R, and mtDNA-2R and the inner primer set of mtDNA-2F, mtDNA-3F, mtDNA-2R, and mtDNA-3R (Table 2). The resulting PCR products were directly sequenced to determine the nucleotide sequences.

### HBV full-length genome sequencing

In order to determine the full-length genome sequence of the HBV DNA, we directly sequenced the partially sequenced fragment A using the primers WA-2L, FA3L, P4AS, B260, B2466, B2830, FA2R, and FA4LAS (Table 2). In order to further sequence the portion missing from the circular HBV DNA (fragment B), we performed nested PCR on the extracted DNA using Prime STAR^®^ GXL polymerase, the primer set S1, S2, AS2, and AS2, and the inner primers S2, S3, AS2, AS3 (Table 2). Fragment B was directly sequenced using primers S4 and AS4 for both directions.

For credible homology analysis, full-length genome sequences were used.

### Genetic analysis and phylogenetic analysis

Genetyx-Mac version 17 (software, Genetyxs Co., Tokyo, Japan) was used for sequence alignment, mutation analysis, mapping analysis, homology analysis, and phylogenetic analysis of the obtained nucleotide sequences. A bootstrap NJ method was used with 1000 re-samplings to produce a phylogenetic tree.

## Results

### Family tree and mitochondria analysis

**Fig 2:**
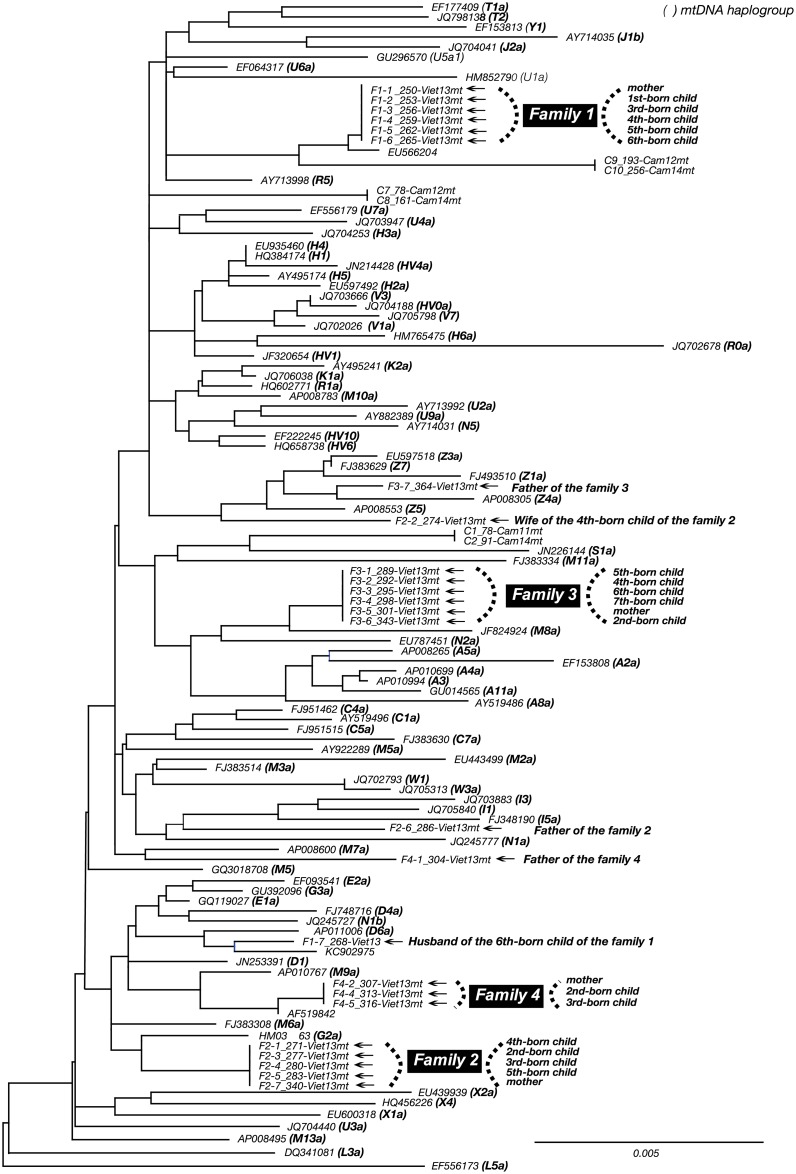
Phylogenetic tree of mitochondrial hyper variable region (HVR) sequences of family members. NJ method was used for construction of the phylogenetic tree, by bootstrap method with one thousand-time resampling. Outgroup is EF556173 (haplogroup L5). Every child was identified having the same sequence as his or her mother, meaning they are in a blood relation.

Mitochondrial analysis was used to determine mother-child relationships [21] as it was necessary to perform the family study on 26 members of four families (Fig 1) and to constitute family trees. Although tissue cells or blood cells are usually used for mitochondrial analysis, in this study we attempted to detect mitochondrial genes in stored blood serum. The region from position 15998 to position 369 of mitochondrial gene was successfully sequenced in both directions in 25 of the family members. In the phylogenetic tree analysis (Fig 2), children had 100% identical sequences to their mothers in each family. Fathers and spouses of children had mitochondrial DNA from different lineages.

### Partial sequences of HBV, phylogenetic analysis among family members and residents

Thirteen out of the 26 family members were positive for HBV DNA: one in family 1, three in family 2, six in family 3, and three in family 4 (Fig 1). Partial sequences were obtained from 35 HBs antigen-positive individuals of residents, and the analysis was performed with a total of 48 subjects.

**Fig 3:**
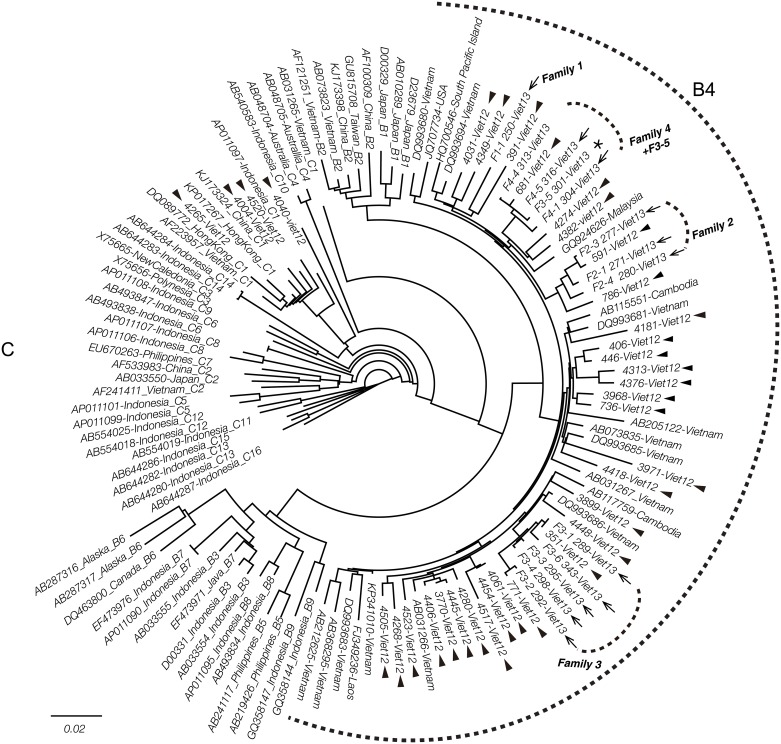
Phylogenetic tree constructed with sequences of HBV polymerase region of obtained isolates. Out of analyzed 48 isolates, forty-four residents and family participants were determined genotype B4 and also four residents were as genotype C1. Obtained residents’isolates are shown by their isolate names with arrow heads. Family member isolates are shown by their family names with arrow. Family 2, 3 and 4 member’s isolates accumulated in respective family’s clusters, except F3-5 marked with an asterisk that was the mother of family 3. Some residents’ isolates were confirmed as neighborhood of family members in each family cluster. Among residents’ isolates, some pair were so close composing a cluster, for example, 4445-Viet12 (28 years old female) and 3770-Viet12 (39 years old male), 4061-Viet12 (30 years old female) and 4454-Viet12 (58 years old female), 736-Viet (27 years old female) and 3968-Viet (25 years old male).

Phylogenetic analysis revealed that 44 isolates were genotype B4 (91.7%) and the remaining four were genotype C1 (8.3%; Fig 3).

In family 1, the sequence of HBV was only found in the F1-1 mother; while the five children participating in the study were all HBsAg and HBV DNA negative (Fig 1A). In families 2, 3, and 4, the intra-family isolates belonged to the same clusters (Fig 3). Among the residents, 591-Viet12 belonged to the same cluster as that of family 2, 351-Viet12 and 771-Viet12 belonged to the same cluster as that of family 3, and 681-Viet12 belonged to the same cluster as that of family 4. On the other hand, the mother F3-5 in family 3 belonged to the family 4 cluster instead of the family 3 cluster. (Fig 3, *)

### Full-length genome analysis of family members and residents

The complete HBV nucleotide sequence was analyzed from 11 family members and 10 residents. Among family members, unfortunately, two samples (F2-4, F3-5) could only be done partial sequencing, but could not be brought to completion to full genome analysis.

**Fig 4:**
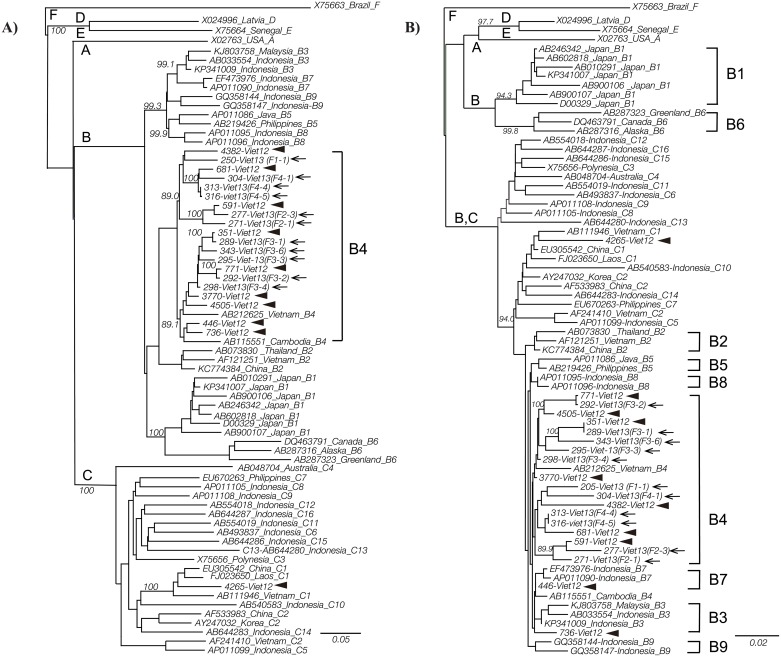
Phylogenetic tree constructed with sequences of HBV complete genome. HBV genotype FX75663 was used for outgroup. An arrow head is indicated for obtained residents isolate and an arrow for family member isolate. (A): Phylogenetic tree of full HBV genome of twenty-one isolates. Obtained all family members isolates were reconfirmed genotype B4 with whole HBV genome analysis. (B): Phylogenetic tree based on the core region sequences of twenty-one whole genome isolates, showed every genotype B4 isolate had a similarity as genotype C at the core region. Consequently, isolates were recombinants, genotype B4/C.

The full nucleotide sequences of HBV genotype B4 found in 20 individuals varied in length of nt3134-3215 genome, and the length of the C1 genotype was nt3206 genome. Additionally, full-length genome phylogenetic analysis also showed that various family members formed clusters with each other (Fig 4A). As in the phylogenetic tree analysis from the partial sequences, some of the isolates in the residents belonged to clusters formed from family members. Looking at the concordance rate in sequence homology analysis, the homology between members of each family was 97.8% for family 2, 96.8–99.3% for family 3, and 98.4–99.4% for family 4. Focusing on details for the four isolates in residents belonging to the same clusters as family members, F2-3 and 591-Viet12 had a 99.3% homology and were both 37-year-old women, F3-1 and 351-Viet12 had a 99.8% homology and were both 30-year-old men, F3-2 and 771-Viet12 had a 99.7% homology and were both 32-year-old women, and F4-4 and 681-Viet12 had a 99.4% homology and were both 25-year-old men.

**Fig 5:**
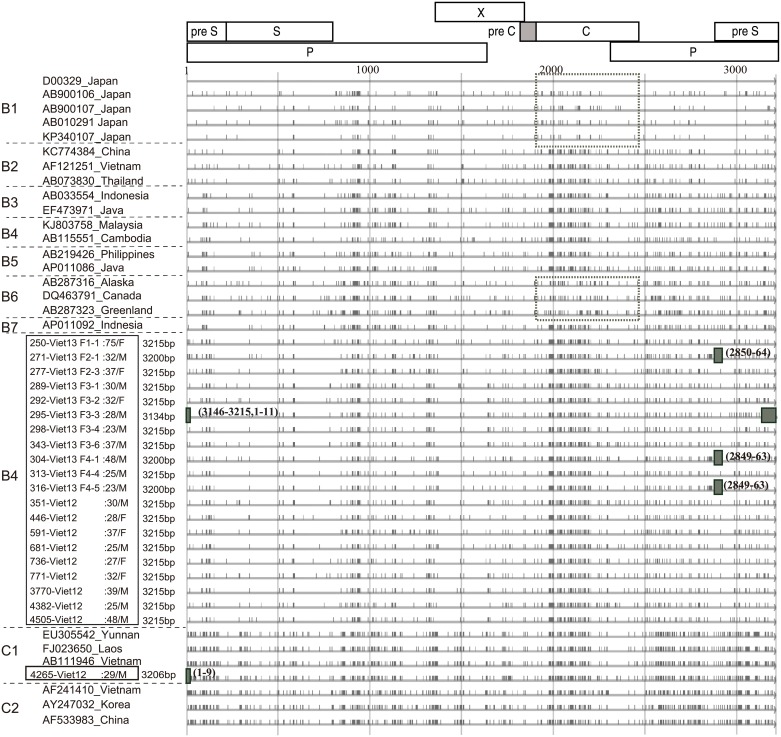
Full genome mapping image with a standard strain D00329, genotype B1. Full HBV genome mapping of obtained twenty-one isolates and known HBV genomes of genotype B and C. Besides the isolate name, age, sex, and nucleotide length are described. At the core region, all genotype B4 isolates have different pattern from genotype B1 or B6 (square in broken line), and almost the same pattern as genotype B2, B3, B5, B7, and C, suggesting they were recombinant at core region strains. Pre-S deletions as shaded squares were recognized in five isolates.

Looking at the mutation sites identified in mapping analysis (Fig 5), the core region of all obtained B4 isolates differed from that of genotypes B1 and B6 by having the same pattern as genotype C. Phylogenetic tree analysis of the core region classified genotypes B1 and B6 into a single group and genotypes B2, B3, B4, B5, B7, B8, and B9 into the same group as genotype C (Fig 4B). The core region sequences of all 20 isolates classified under B4 matched genotype C, and so genotype B4 was found to be a recombinant of genotype C (genotype B4/C). What is more, the core region of 446-Viet12 was similar to genotype B7 and the core region of 736-Viet12 was similar to genotype B3.

As indicated by the mapping analysis (Fig 5), there were five isolates (23.8%) with a pre-S deletion. The deletion was 15 bp of nt2850-2864 in F2-1, 81 bp of nt3146-3215, 1–11 in F3-3, 15 bp of nt2849-2863 in F4-1, 15 bp of nt2849-2863 in F4-5, and 9 bp of 1–9 in 4265-Viet12.

### Promoter mutation

**Fig 6:**
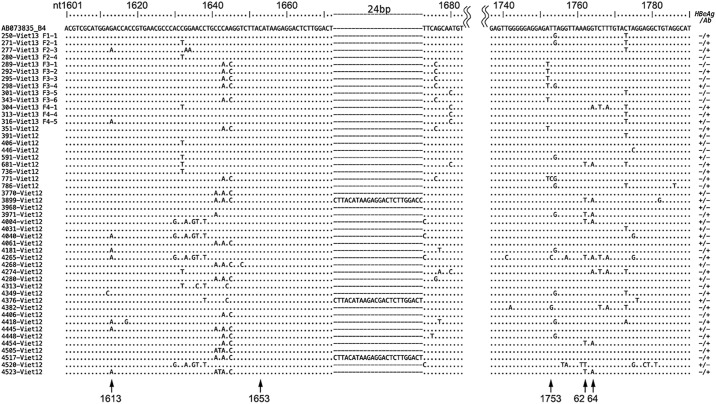
Partial nucleotide sequences from nt1601 to nt1790 including core promoter of 48 obtained isolates. Each genotype is shown besides each isolates name. The upper line sequence is AB073835, genotype B4, as a standard. HBeAg and HBeAb status are described at the right side of figure. In forty-four genotype B4 isolates, mutation rate at nt1613 was 13.6%, at nt1753 was 4.5%, at nt1762/64 was 11.4%. Insertions were detected in three isolates with 24 base starting from nt1673, just middle in the promoter, in X region.

Six out of the 44 isolates of HBV genotype B4 (13.6%) had a promoter mutation in G1613A (Fig 6). There were no C1653T mutations, two (4.5%) T1753V mutations, five (11.4%) double mutations of A1762T/G1764A, and three (6.8%) double mutations of C1766T/T1768A. Three (6.8%) isolates (3899-Viet12, 4376-Viet12, 4517-Viet12) had a 24 bp insertion from nt1673 in the X region.

## Discussion

Southeast Asia remains a region with a high rate of HBV infection, within which Vietnam is located. Vietnam has been an area with a high prevalence of HBV, with over half the population having a history of HBV infection [9–11]. It is reported that many cases of liver diseases including chronic hepatitis in Vietnam are associated with high prevalence of HBV instead of low prevalence of HCV. Deaths from HCC ranks the first in cancer deaths in Vietnam[17], HCC cases in Vietnam mainly attribute its origin to HBV [22]. Especially, there is a high prevalence of HBV genotype B, with genotype B4 constituting 74% of cases and C1 constituting 22% of cases in Hanoi [10, 23–26]. A multicenter study in Vietnam showed that genotype B4 was dominant, with 82.6% prevalence compared to 14.6% for C1 [27]. In the present study HBV genotype B4 constituted 91.7% of cases.

While investigating transmission pathways through phylogenetic trees, all five children of the HB carrier mother in family 1 were HBs antigen-negative and HBV DNA negative. The mother-child relationship of the five was confirmed through mitochondrial analysis. No evidence of mother to child infection was found in this family.

In family 2, neither the father nor the mother was an HB carrier. Out of all the isolates obtained from the three children, F2-3 in the second-born child belonged to the same cluster as F2-1 and F2-4 in the other two siblings. Furthermore, sequence homology was high at 99.3% between F2-3 and a resident 591-Viet 12, who was of the same age and sex. We inferred that there was a high likelihood of horizontal transmission between these two individuals.

In family 3, the mother F3-5 was HBsAg and HBsAb negative. However, she was strongly positive for HBcAb at 11.3 IU/ml with the CLEIA method more over to detection of HBV DNA. In phylogenetic tree analysis, the isolate in the mother F3-5 belonged to the cluster of family 4 rather than of family 3. On the other hand, the five children F3-1, F3-2, F3-3, F3-4, and F-3-6 formed a single separate cluster with a high sequence homology of 98.5–99.2%. In addition, even higher homology rates were identified between F3-1 and a resident 351-Viet12, and also between F3-2 and a resident 771-Viet12, both were of the same age and sex. We strongly inferred horizontal transmission between siblings and members of the same generation.

In family 4, the father and children belonged to the same cluster (with a sequence homology of 98.4% and 98.9%, respectively), but the phylogenetic tree shows that the father’s F4-1 isolate was similar to resident isolate 681-Viet12. The homology between siblings F4-5 (23 years old) and F4-4 (25 years old) was high at 99.4%. By hypothesizing that in family 4, transmission from the father to the two children occurred during their infancy, a calculation of the mutation rate between the father F4-1 and the children F4-4, F4-5 resulted in 5.5×10^−4^ to 7.3×10^−4^ /sites/year. This was a larger value than the reported HBV mutation rate of 1.4–3.2×10^−5^ to 1.44×10^−4^/site/year [28–31].

In particular, in the four sets of people at the same age and sex among whom we found high sequence homology discussed above, the calculated mutation rate hypothesizing transmission during infancy resulted in 6.8×10^−5^ to 2.5×10^−4^ /site/year. As a result, we found that the pairs with isolates from residents yielded values much closer to the reported HBV mutation rate which was considered to be a piece of evidence for horizontal transmission.

The possible limitations of this study had been recognized. First in the family study only four families could participate, thereby might cause sampling bias. The actual situation is that the participation in a study is very precious behavior because blood-sampling tests are not yet common in rural areas in Vietnam.

Second, due to the technical efficiency of PCR for HBV sequencing, we could not reveal full genome sequence of some persons. This also might cause sampling bias.

Some pairs of residents had close isolates on a cluster by phylogenetic tree constructed with partial sequences, but could not be shown as full-genome sequences. In further study, precise investigations are needed on geographical location, links between these individuals, analysis not only from the nuclear family but extended family

As far in the results of this study, the isolates of family members were generally similar to each other, but we found that the sequence homology was even greater with respect to some isolates in residents. Since identified pairs were of the same age and sex, we strongly inferred that there was horizontal transmission. The phylogenetic tree and homology analysis of the HBV full genome suggested the probability and its possibility of horizontal transmission not only intra-family nor vertical transmission but within cohorts of the same generation in the population.

HBV genotype B4 was dominant in the current survey, and all isolates were found to be genotype B4 or C1. Compared to genotype C, genotype B is thought to be a lower risk of HCC [13, 14]. And it is known that the sub-genotype of genotype B4 has experienced core region recombination with genotype C [32–34]. HBV genotype B4 in the current study did have core region recombination with genotype C, and so it is conceivable that higher risks of HCC could arise.

On the other hand, the pathology is also affected by HBV genome mutations, and it has been recently shown that genetic characteristics of HBV of promoter mutations and pre-S region deletions are strongly associated with HCC risk [16, 18, 19, 35–40].

Reported promoter mutations are G1613A, C1653T, and T1753V single mutations and A1762T/G1764A double mutations. Takahashi et al. showed a correlation between HCC and promoter mutations, pre-S2 region mutations and deletions, X region insertions, and the like [18]. Tatsukawa et al. reported that single G1613A mutation was associated with future emergence of HCC [37]. Yang et al. noted that when HCC risk was calculated based on meta-analysis of HBV mutations and HCC, nt1762/1764 and pre-S deletions each had RR>1 [19].

GenBank has 31 registered strains of the full genome sequence of HBV genotype B4 (as of October 2016). But looking at the promoter region mutations, G1613A mutation was identified in 10.3% of these 31 strains, C1653T was 0%, T1753V was 3.2%, A1762T/G1764A was 16.1%, and C1766T /T1768A was 3.2%. Pre-S region deletions were found in two strains at the rate of 6.5%.

In the 44 obtained isolates of genotype B4, mutations mentioned above were identified at the rate of 13.6%, 0%, 4.5%, 11.4%, and 6.8%, respectively. Thus, in the genotype B4 existing strains and the isolates we obtained, nt1613 mutation was common and nt1653 mutation was rare. In addition, the 24 bp insertion from nt1673 at X region, which also in the promoter region, which is not often reported, was found to be 6.8% among the obtained isolates. Pre-S region deletion was found in 20.0% of cases, which is higher than in the B4 strains searched in GenBank.

Since full genome registration of HBV genotype B4 in GenBank is yet not plentiful in number, the 21 full genome sequences obtained in this study can contribute to future studies.

HBV genotype B4 in the current residents and families had recombination at core region, genotype B4/C, with genotype C. Promoter point mutations and insertions at X region and pre-S region deletions were found, and so we inferred an association with higher risks of HCC. As a result, the individuals with HBV having genetic mutations as identified in the present study should receive proper follow-up regarding the development of liver diseases and HCC.

## Abbreviations

HBcAb: Hepatitis B virus core antibody
HBsAg: Hepatitis B virus S antigen
HBV: hepatitis B virus
HCC: hepatocellular carcinoma
HCV: Hepatitis C virus
HVR: hyper variable region
WHO: World Health Organization
